# Predictors of osteoradionecrosis following irradiated tooth extraction

**DOI:** 10.1186/s13014-021-01851-0

**Published:** 2021-07-14

**Authors:** Szu Ching Khoo, Syed Nabil, Azizah Ahmad Fauzi, Siti Salmiah Mohd Yunus, Wei Cheong Ngeow, Roszalina Ramli

**Affiliations:** 1 Department of Oral and Maxillofacial Surgery, Faculty of Dentistry, Universiti Kebangsaan Malaysia, Jalan Raja Muda Abdul Aziz, 50300 Kuala Lumpur, Malaysia; 2 Department of Craniofacial Diagnostics and Biosciences, Faculty of Dentistry, Universiti Kebangsaan Malaysia, Jalan Raja Muda Abdul Aziz, 50300 Kuala Lumpur, Malaysia; 3 Department of Oral and Maxillofacial Clinical Sciences, Faculty of Dentistry, University of Malaya, Kuala Lumpur, 50603 Malaysia

**Keywords:** Osteoradionecrosis, Dental extraction, Post radiotherapy, Predictors

## Abstract

**Background:**

Tooth extraction post radiotherapy is one of the most important risk factors of osteoradionecrosis of the jawbones. The objective of this study was to determine the predictors of osteoradionecrosis (ORN) which were associated with a dental extraction post radiotherapy.

**Methods:**

A retrospective analysis of medical records and dental panoramic tomogram (DPT) of patients with a history of head and neck radiotherapy who underwent dental extraction between August 2005 to October 2019 was conducted.

**Results:**

Seventy-three patients fulfilled the inclusion criteria. 16 (21.9%) had ORN post dental extraction and 389 teeth were extracted. 33 sockets (8.5%) developed ORN. Univariate analyses showed significant associations with ORN for the following factors: tooth type, tooth pathology, surgical procedure, primary closure, target volume, total dose, timing of extraction post radiotherapy, bony changes at extraction site and visibility of lower and upper cortical line of mandibular canal. Using multivariate analysis, the odds of developing an ORN from a surgical procedure was 6.50 (CI 1.37–30.91, *p* = 0.02). Dental extraction of more than 5 years after radiotherapy and invisible upper cortical line of mandibular canal on the DPT have the odds of 0.06 (CI 0.01–0.25, *p* < 0.001) and 9.47 (CI 1.61–55.88, *p* = 0.01), respectively.

**Conclusion:**

Extraction more than 5 years after radiotherapy, surgical removal procedure and invisible upper cortical line of mandibular canal on the DPT were the predictors of ORN.

## Introduction

Radiotherapy (RT) is an effective treatment modality for head and neck cancer. It is used for curative intent, or adjuvant with surgery or as a palliative treatment. It has many complications; among those is osteoradionecrosis (ORN). ORN is defined as “an exposed necrotic bone for more than 3 months with no signs of tumour recurrence in the previously irradiated area” [1, 2].

ORN occurs spontaneously or may be preceded by trauma or surgery [3, 4]. Dental extraction is recognized as one the most important risk factor of ORN [5]. The incidence of ORN following a dental extraction ranged from 0 to 7% [2, 6–8]. Among the known risk factors of a post-extraction ORN reported in the literature were high total dose of more than 60 Gy and the area of extraction was in the target volume [4].

Dental panoramic tomograph (DPT) is the initial radiographic examination usually performed to examine the ORN changes [9]. Literature described radiographic changes of the irradiated bone [10, 11] as well as the ORN [9, 12, 13]. The irradiated (post RT) bone radiographic changes included (1) widening of the periodontal ligament space, (2) bone sclerosis, (3) periodontal disease-like bone loss, and (4) bone resorption [10].

One intriguing anatomical feature of the mandible is the mandibular canal. The mandibular canal change following RT has not been very well described. Mandibular canal (MC) walls are composed of a coalescence of trabecular bone [14]. Its radiographic appearance included a radiolucent zone lined by two borders: the superior and inferior borders. The visibility of the superior and inferior borders of mandibular canal in DPT has been studied in the normal population [15–19]. In relation to this study, the visibility or invisibility of the MC are related to the change in bone activity around the neurovascular bundles [14, 20, 21]. In ORN, the osteoclastic activation and attenuation of osteoblastic function results in loss of cortical outlines and trabecular bone density [9]. Whether or not there is an association between ORN change and MC visibility has not been explored.

Tooth extraction post RT sometimes is inevitable despite comprehensive dental care prior to it. There were reported cases of progression of ORN despite current therapeutic measures [3, 22].

This study aimed to investigate the association between the dental and radiographic related study factors and ORN following a dental extraction. Following that, the predictors from these two clinical components were assessed.

## Materials and methods

This study was approved by the Universiti Kebangsaan Malaysia Research Ethics Committee (UKM 41 PPI/111/8/JEP-2018-340), the Medical Ethics Committee of Faculty of Dentistry University Malaya (DF OS1913/0051(L)) and University Malaya Medical Centre (UMMC) Medical Research Ethics Committee (MREC ID NO: 2019611-7512). This retrospective record analysis was conducted in two university hospitals in Malaysia: Oral and Maxillofacial Surgery Clinic, Universiti Kebangsaan Malaysia Medical Centre (UKMMC) and Faculty of Dentistry, University of Malaya (FDUM) from 1st July 2018 until 31st March 2020. Records of patients who had dental extraction performed between 2nd August 2005 to 21st October 2019 in the oral and maxillofacial clinic in both centres were reviewed.

The inclusion criteria were:

1. A history of head and neck radiotherapy. The radiotherapy intention could be either curative or palliative.
2. Dental extraction was performed after radiotherapy.
3. The timing of tooth extraction could be as early as the first day post radiotherapy until as long as the patient lives.

The exclusion criteria were:

1. A history of anti-resorptive medication or bisphosphonate
2. A known case of recurrence or metastatic tumor to the ORN site

### Data collection

The data collected included the clinical and radiographic factors.

#### Clinical data

The medical records of the patients were reviewed. The independent and dependent variables collected included.

##### Independent variables

1. Demographic, health and habits, tumour, oral hygiene status and number of teeth extracted in a patient. Oral hygiene status was categorized into good, moderate or poor based on the assessment of the first OPG taken. If there was no caries in the initial DPT, it was considered as good oral hygiene; 1 or 2 caries meant moderate oral hygiene and 3 or more caries meant poor oral hygiene [23].
2. Radiation factors, i.e. target volume and total dose. In determining the target volume, the site of dental extraction was compared with the planning target volume area. For radiotherapy planning of the 2D RT, the target volume was demarcated in the X-ray film by line X1, X2 (vertical) and Y, Y2 (horizontal). For the site of dental extraction that was in the demarcated area was recorded as “in the target volume”. For the computerized method, the radiotherapy simulation plan (CT scan) was reviewed using a computer. For the site of dental extraction that was in the planning target volume demarcated area, it was recorded as “in the target volume”. For the site of dental extraction that was out of the demarcated area, it was recorded as “not in the target volume”. Fractionation was not included for analysis.
3. Dental extraction characteristics.
  Information in relation to tooth type, tooth pathology, procedure, operator, primary closure, antibiotic post-extraction, teeth within target volume, total dose, time of extraction post radiotherapy.
4. Radiographical factors, i.e. visibility of the mandibular canal, bony changes in mandible, widening of periodontal ligament space and lamina dura continuity.

##### Dependent variable/outcome

1. ORN
  ORN for the purpose of this study is defined “clinically exposed bone for a duration of three months or more following a dental extraction” [2, 24] and with no history of anti-resorptive medication or bisphosphonate.

#### Radiographic data

1. The dental panoramic tomogram (DPT) record was searched in the Picture Archiving and Communication System (PACS) in UKMMC. The DPT machine used was the Vatech PaX-Reve3D with 80 kilovolt peak (kVp), 10 milliampere (mA) seconds mode and 1.3 magnification ratio. All the digital images were reviewed using the Hp computer (model: Compaq dc7800 Convertible Minitower) with image displayed on a 13.5-inch monitor in the Oral and Maxillofacial Clinic, UKMMC. The DPT film that was available in the patient’s record was digitalized in the Radiology Department, UKMMC using the Microtek Medi-6000 Plus scanner. The DPT film which had poor quality such as trace of being folded multiple times and/or image blurriness or deterioration due to old age was excluded as the assessment was deemed impossible. In the FDUM, the DPT was taken with either the Kodak 9000C 3D (Rochester, NY) or the J Morita machine. The Kodak 9000C 3D has 60–90 kVp of tube voltage, 2–15 mA of tube current and 1.27 magnification ratio. The J Morita machine has 60–90 kVp, 1–10 mA and 1.3 magnification ratio. The DPT was reviewed using the Siemens *syngo*® XS Imaging Viewer.

### Assessment method

All the DPTs were assessed by one examiner. Prior to the assessment, the examiner had two calibration sessions with the oral and maxillofacial radiologist. For inter-observer and intra-observer reliability assessment, ten pre-extraction post radiotherapy DPT were assessed. The criteria being assessed were bony changes and visibility of mandibular canal. The inter-observer and intra-observer reliability were assessed with Cohen’s kappa. The inter-observer reliability was between 0.4 and 0.9 while the intra-observer reliability was between 0.41 and 0.9. The DPT was then assessed for bone changes and mandibular visibility.

#### Bone changes

**Fig. 1:**
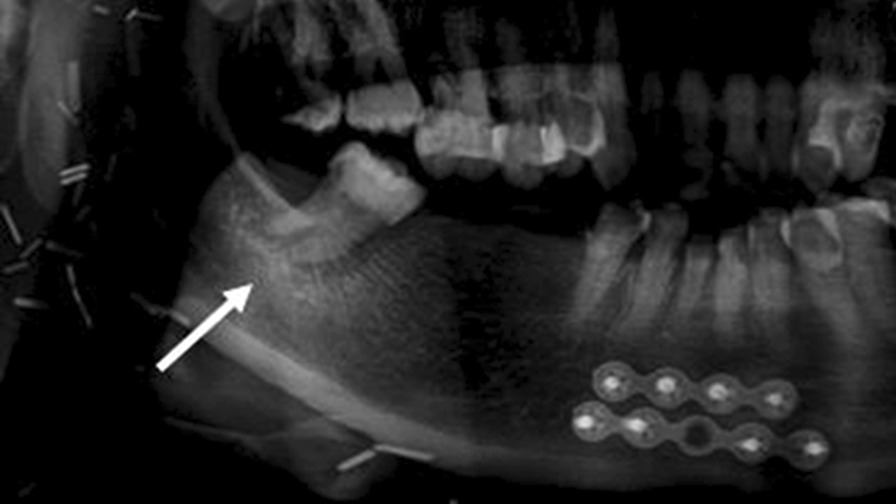
Bony sclerosis in relation to tooth 47

**Fig. 2:**
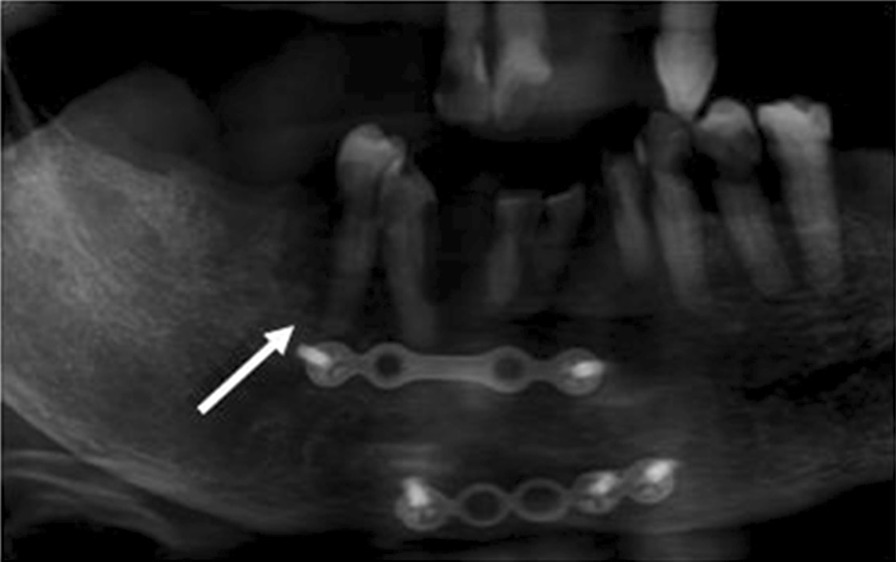
Bony resorption in relation to tooth 44

**Fig. 3:**
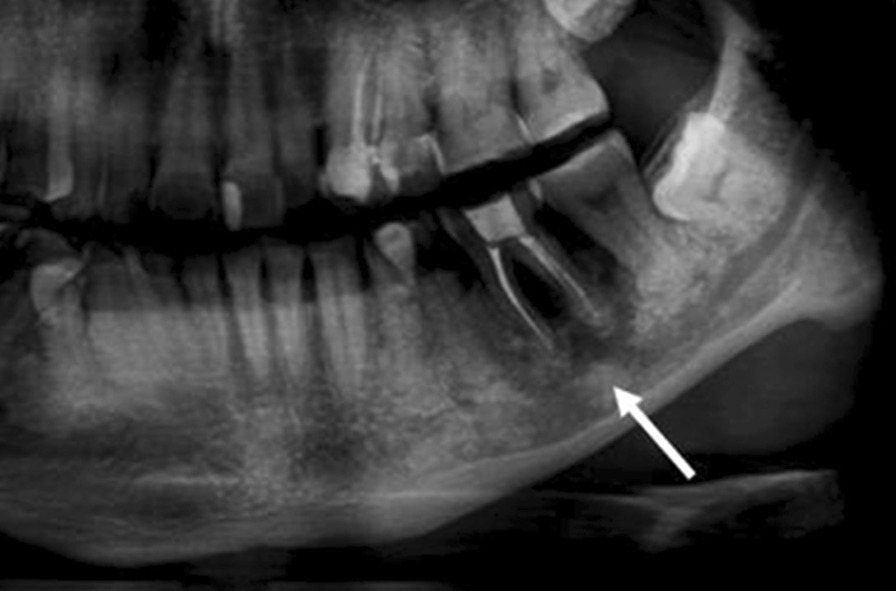
Mixed radiopaque-radiolucent lesion in relation to tooth 36

1. Widening of the periodontal ligament space (WPLS). The definition of WPLS is “increased width of the periodontal ligament space greater than 0.5 mm along the entire length of the tooth root without a specific epicenter and adjacent bone destruction, provided that it is unrelated to apical periodontitis secondary to caries or periodontitis [10]”.
2. Irregularity, interruption or loss of lamina dura, provided that it is unrelated to apical periodontitis secondary to caries or periodontitis.
3. Bone sclerosis. The definition is “a region of increased bone density resulting from an increased number of bone trabeculae [10]” (Fig. 1).
4. Bone resorption. The definition is “a region of decreased bone density resulting from a decreased number of trabeculae [10]” (Fig. 2).
5. Mixed radiopaque-radiolucency [25] (Fig. 3).

The area of bone changes was recorded adjacent to the site of dental extraction.

#### Mandibular canal visibility

**Fig. 4:**
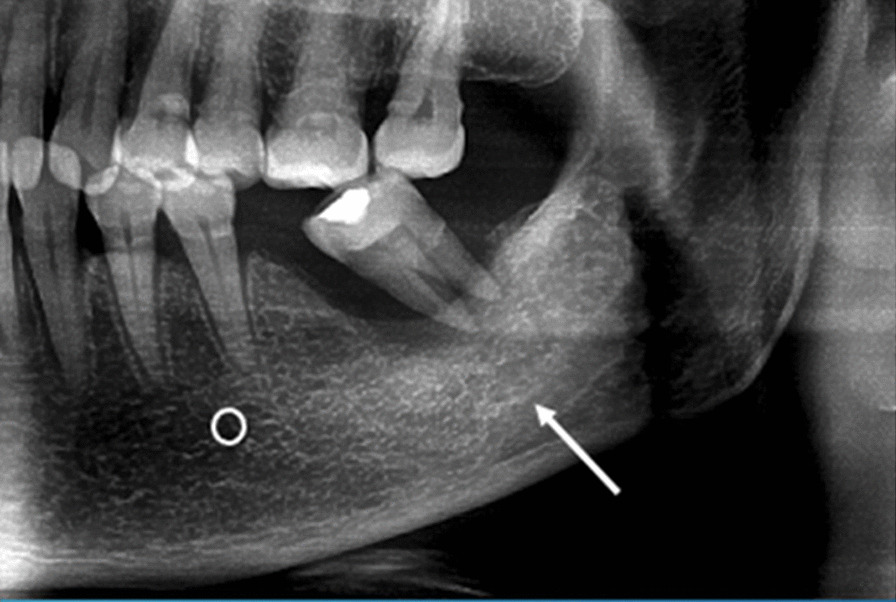
Visible upper cortical line of the left mandibular canal

For the assessment of mandibular canal visibility, the ramus and body of mandible on left and right were included as the area for inspection. We categorized ‘visible’ and ‘invisible’ of the upper and lower cortical line of the mandibular canal as below:

Visible: clear continuous line or faint or interrupted line (Fig. 4).

**Fig. 5:**
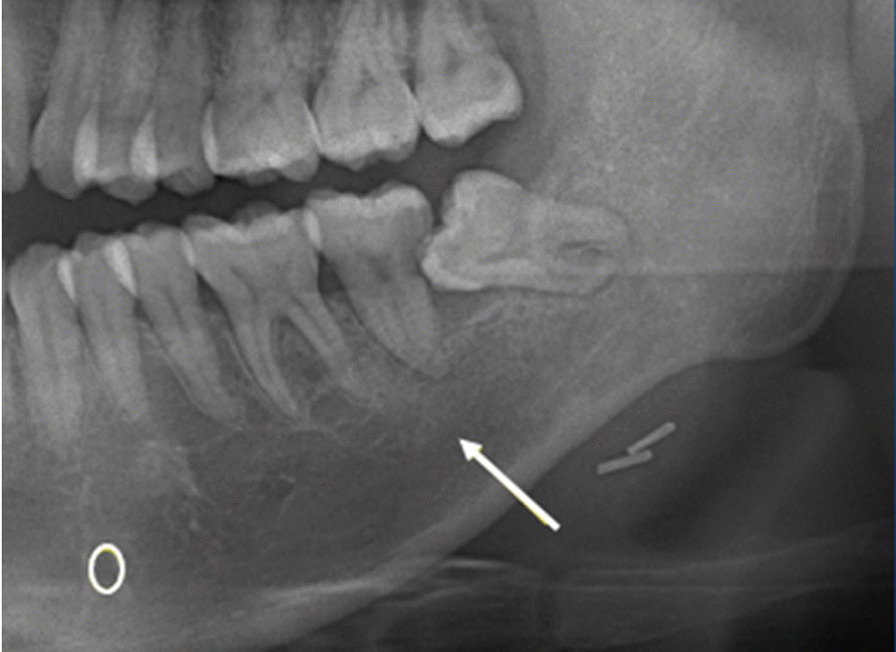
Invisible upper cortical line of the left mandibular canal

Invisible: no line or mark that could be observed at all (Fig. 5).

For canine to second molar extraction, the body of mandible was inspected and for third molar extraction, the ramus was also included. Bony changes and mandibular canal visibility were reported based on the site of extraction.

### Statistical analysis

Data entry and analysis was conducted using the IBM SPSS Statistics version 20.0 (Armonk, NY: IBM Corp.). Descriptive analysis was shown as frequencies, percentage, mean and standard deviation (SD), where appropriate. Pearson’s chi-square and Fisher’s exact test were used to determine the association between the clinical and radiographical factors and ORN post dental extraction. A significance level was set at *p* < 0.05.

Binary logistic regression analysis was employed to examine the association between the independent (predictors) and dependent (outcome) variables. The factors that reached significance level (*p* < 0.25) from the univariate analysis was included into the model [26].

The *p* value of 0.25 was selected based from the Wald test from the logistic regression. The traditional level of 0.05 fails to recognize variables that are equivalently crucial [27, 28]. In addition, variables with *p* > 0.25 but with clinical significance were also included.

The method used was backward conditional [29]. Quality of the model was assessed for goodness of fit using the Hosmer–Lemeshow test and the classification table. Obtaining a non-significant result in the Hosmer–Lemeshow test would indicate that the model was well calibrated, and therefore was considered as fit. In the classification table, models with percentage of overall correctly classified closer to 100% were considered as more accurate.

## Results

### Patient characteristics

Seventy-three patients who had dental extraction post radiotherapy to the head and neck were included. Of the 73 patients, 16 had ORN post dental extraction. Of the 389 teeth extracted, 32 sockets developed ORN. The prevalence of ORN were 21.9%.

There were 41 men and 32 women with median age of 47 years (IQR = 38.5–55.0 years). They had 389 extractions (mandible: n = 198, maxilla: n = 191) at a mean year of 9.02 ± 6.57 years (range 0.4–47.3 years) after radiotherapy (Table 1).

**Table 1:** Demographic, health and habit factors, tumour, radiation, surgery involving the jaws, oral hygiene in patients who had dental extraction post radiotherapy to the head and neck (N = 73)

Variable	Category	n (%)
Age	47 (38.5–55.0)	
Gender	Male	41 (56.2%)
	Female	32 (43.8%)
Medical illness	Hypertension	23 (31.5%)
	Endocrine-related (DM, Thy)	14 (19.3%)
	No other medical illness	31 (42.4%)
	Others	4 (5.5%)
	No data	1 (1.4%)
Smoking status	Smoker	4 (5.5%)
	Non-smoker	59 (80.8%)
	No data	10 (13.7%)
Alcohol intake status	Drinker	3 (4.1%)
	Non-drinker	53 (72.6%)
	No data	17 (23.3%)
Tumour site	Oral	14 (19.2%)
	Oropharynx	6 (8.2%)
	Nasopharynx	40 (54.8%)
	Larynx	5 (6.8%)
	Other sites	8 (11.0%)
Clinical stage (AJCC)	I	5 (6.8%)
	II	13 (17.8%)
	III	18 (24.7%)
	IV	18 (24.7%)
	No data	19 (26.0%)
Radiation plan	RT only	27 (37.0%)
	Concurrent chemoradiotherapy	35 (47.9%)
	No data	11 (15.1%)
Type of radiotherapy	2D RT	35 (47.2%)
	3D CRT	10 (13.9%)
	IMRT	3 (4.2%)
	No data	25 (34.7%)
Pre-radiotherapy surgical intervention	Mandibular surgery	10 (13.7%)
	Maxillectomy	4 (5.5%)
	Without surgical intervention	55 (75.3%)
	No data	4 (5.5%)
Oral hygiene status	Good	6 (8.2%)
	Moderate	14 (19.2%)
	Poor	44 (60.3%)
	No data	9 (12.3%)
No of teeth removed in a patient	≤ 3	40 (54.8)
	> 3	33 (45.2)
Other late toxicity of RT		
Xerostomia	Yes	46 (63.0%)
	No	10 (13.7%)
	No data	17 (23.3%)
Trismus	Yes	15 (20.5%)
	No	51 (69.9%)
	No data	7 (9.6%)

*DM* diabetes mellitus, *Thyr* hyper/hypothyroidism, *2D RT* two-dimensional radiotherapy planning, *3D CRT* three dimensional conformal RT, *IMRT* intensity modulated radiation therapy

The most frequent malignancy of the head and neck was nasopharyngeal carcinoma (NPC) (n = 40, 54.8%), followed by squamous cell carcinoma (SCC) of the tongue (n = 11, 15.1%). Majority (n = 36, 49.4%) had Stage III or IV disease. Median total dose for 64 patients was 70 Gy (IQR = 61–70 Gy). All patients except three (n = 61, 83.6%) received total dose of 60 Gy and above (Table 1).

Most patients were shown with poor oral hygiene (60.3%). The median of tooth extraction per patient was 3.0 (IQR 4.5). Frequency of patients who had three of less tooth extractions was 40 (54.8%) and more than three teeth removed was 33 (45.2%) (Table 1).

In relation to late toxicity of radiotherapy, xerostomia was shown to be profound, it involved 63% of these patients (Table 1).

### Dental extraction characteristics

The teeth that were commonly extracted were the upper molars (n = 90, 23.1%), lower incisors/canines (n = 86, 22.1%), and lower molars (n = 75, 19.3%). The most common reasons for a dental extraction were retained roots (n = 178, 45.7%), periapical periodontitis or abscess (n = 145, 37.3%) and chronic periodontitis with tooth mobility (n = 42, 10.8%). Dental extraction characteristics were shown in Table 2.

**Table 2:** Dental extraction characteristics (N = 389)

Variable	Category	n (%)
Tooth type	Lower incisor/canine	86 (22.1%)
	Upper incisor/canine	55 (14.1%)
	Lower premolar	42 (10.8%)
	Upper premolar	41 (10.5%)
	Lower molar	75 (19.3%)
	Upper molar	90 (23.1%)
Tooth pathology	Periapical periodontitis related	145 (37.3%)
	Caries-related	178 (45.7%)
	Perio-related	42 (10.8%)
	Mixed caries-perio related	8 (2.1%)
	Others	4 (1.1%)
	No data	12 (3.1%)
Procedure	Simple extraction	259 (66.6%)
	Surgery	125 (32.1%)
	No data	5 (1.3%)
Operator	Dental Officer	188 (48.3%)
	Postgraduate	81 (20.8%)
	Specialist	113 (29.0%)
	No data	7 (1.8%)
Primary closure	Yes	198 (50.9%)
	No	186 (47.8%)
	No data	5 (1.3%)
Antibiotic post-extraction	One antibiotic	255 (65.6%)
	Multiple antibiotics	84 (21.6%)
	No antibiotic prescribed	44 (11.3%)
	No data	6 (1.5%)
Teeth within target volume	Yes	107 (27.5%)
	No	107 (27.5%)
	No data	175 (45.0%)
Total dose	Below 60 Gy	122 (31.4%)
	60 Gy and above	95 (24.4%)
	No data	172 (44.2%)
Time of extraction post radiotherapy	3 months to 1 year	13 (3.3%)
	1–5 years	103 (26.5%)
	> 5 years	273 (70.2%)

### Radiographic characteristics

A total of 159 extracted mandibular teeth were assessed for radiographic bony changes and mandibular canal visibility. The extracted teeth comprised 70 anterior teeth (incisors and canines), 37 premolars and 52 molars.

#### Bone change

Majority (n = 73, 45.9%) showed no bony changes at the extraction site, followed by presence of bony sclerosis (n = 58, 36.5%) and mixed radiopaque-radiolucent changes (n = 19, 11.9%). 5.7% showed bony resorption.

#### Visibility of the upper and lower cortical line of the mandibular canal

DPT of 107 mandibular teeth (excluding the lower incisors) were evaluated for the visibility of the upper and lower cortical lines of the mandibular canal.

For the upper cortical line (UCL), 53 (49.5%) lines were not visible; others were visible with various visibilities: interrupted lines (n = 48, 44.8%) and faint lines (n = 6, 5.6%).

For visibility of the lower cortical line (LCL) of the mandibular canal, 15 (14.0%) lines were not visible and the rest were visible, i.e. interrupted (n = 82, 76.6%), followed by faint lines (n = 10, 9.3%).

#### Periodontal ligament space change and lamina dura continuity

Of the 389 teeth, only 140 teeth (36%) were assessed for widening of periodontal ligament space and lamina dura continuity. The remaining 249 teeth were excluded due to presence of periapical lesion which were due to caries or periodontitis (n = 144, 37%), absence of pre-extraction DPT (n = 86, 22.1%), and poor image quality (n = 19, 4.9%). For widening of the periodontal ligament space (N = 140), 138 teeth (98.6%) did not have widening of periodontal ligament space while two teeth (1.4%) had widening of periodontal ligament space. For the lamina dura continuity (N = 140), all teeth (100%) had interrupted lamina dura.

### Univariate analyses

#### Association between dental-related factors and ORN post dental extraction

The association between dental-related factors and ORN was explored through univariate analyses. The results are shown in Table 3 below. Seven variables that showed significant results were: (1) tooth type (*p* < 0.001) (2) tooth pathology (*p* = 0.001) (3) dental extraction procedure (*p* < 0.001) (4) primary closure (*p* = 0.029) (5) within target volume (*p* < 0.001) (6) total dose (*p* < 0.001) (7) timing of extraction post radiotherapy (*p* = 0.001).

**Table 3:** The association between dental-related factors and ORN post dental extraction (N = 389)

Variable	All extracted teeth	No ORN, n (%)	ORN, n (%)	*p* value
**Tooth type**				
Upper single-rooted	56 (14.4%)	56 (15.7%)	0 (0.0%)	
Upper double-rooted	135 (34.7%)	122 (34.2%)	13 (40.6%)	< 0.001^a^*
Lower single-rooted	85 (21.9%)	84 (23.5%)	1 (3.1%)	
Lower double-rooted	113 (29.0%)	95 (26.6%)	18 (56.2%)	
**Tooth pathology**				
Periapical periodontitis related	145 (37.3%)	136 (39.0%)	9 (32.1%)	0.001^a^*
Caries-related	178 (45.7%)	164 (47.0%)	14 (50.0%)	
Perio-related	42 (10.8%)	42 (12.0%)	0 (0.0%)	
Mixed caries-perio related	8 (2.1%)	5 (1.4%)	3 (10.7%)	
Others	4 (1.1%)	2 (0.6%)	2 (7.1%)	
No data	12 (3.1%)			
**Procedure**				
Simple extraction	259 (66.6%)	249 (69.9%)	10 (35.7%)	< 0.001^b^*
Surgery	125 (32.1%)	107 (30.1%)	18 (64.3%)	
No data	5 (1.3%)			
**Operator**				
Dental officer/postgraduate	269 (69.2%)	252 (71.0%)	17 (63.0%)	0.379^b^
Specialist	113 (29.0%)	103 (29.0%)	10 (37.0%)	
No data	7 (1.8%)			
**Primary closure**				
Yes	198 (50.9%)	178 (50.0%)	20 (71.4%)	0.029^b^*
No	186 (47.8%)	178 (50.0%)	8 (28.6%)	
No data	5 (1.3%)			
**Antibiotic post-extraction**				
Antibiotics prescribed	339 (87.1%)	315 (88.7%)	24 (85.7%)	0.547^a^
No antibiotic prescribed	44 (11.3%)	40 (11.3%)	4 (14.3%)	
No data	6 (1.5%)			
**Within target volume**				
Yes	107 (27.5%)	87 (45.3%)	20 (90.9%)	< 0.001^b^*
No	107 (27.5%)	105 (54.7%)	2 (9.1%)	
No data	175 (45.0%)			
**Total dose**				
Below 60 Gy	122 (31.4%)	122 (62.6%)	0 (0.0%)	< 0.001^b^*
60 Gy and above	95 (24.4%)	73 (37.4%)	22 (100.0%)	
No data	172 (44.2%)			
**Time of extraction post radiotherapy**				
3 months to 1 year	13 (3.3%)	13 (3.6%)	0 (0.0%)	0.001^a^*
1–5 years	103 (26.5%)	85 (23.8%)	18 (56.2%)	
> 5 years	273 (70.2%)	259 (72.5%)	14 (43.8%)	

*p < 0.05

^a^Fisher’s exact test

^b^Pearson chi-square test

#### Association between radiographic factors and ORN post dental extraction

Three variables, bone change of the mandible, visibility of the upper cortical line and lower cortical line of mandibular canal were included in the tests. The results are shown in Table 4. All three variables showed statistically significant association with ORN.

**Table 4:** The association between bony changes in mandible and mandibular canal visibility with ORN post dental extraction

Variable	All extracted teeth, n (%)	Teeth with no ORN, n (%)	Teeth with ORN, n (%)	*p* value
Bony changes				
Sclerosis	58 (36.5%)	54 (37.5%)	4 (26.7%)	0.022^a^*
Resorption	9 (5.7%)	7 (4.9%)	2 (13.3%)	
Mixed radiopaque-radiolucency	19 (11.9%)	14 (9.7%)	5 (33.3%)	
No change	73 (45.9%)	69 (47.9%)	4 (26.7%)	
Mandibular canal UCL visibility^c^				
Visible	54 (50.5%)	50 (54.3%)	4 (26.7%)	0.047^b^*
Invisible	53 (49.5%)	42 (45.7%)	11 (73.3%)	
Mandibular canal LCL visibility^c^				
Visible	92 (86.0%)	82 (89.1%)	10 (66.7%)	0.035^a^*
Invisible	15 (14.0%)	10 (10.9%)	5 (33.3%)	

*UCL* upper cortical line, *LCL* lower cortical line

^a^Fisher’s exact test

^b^Pearson chi-square test

^c^LOWER incisors were not applicable to mandibular canal visibility assessment (N = 107)

### Multivariate analysis

Predictors of ORN post dental extraction were ascertained from the multivariate analysis. The process was explained earlier in Methods. Fourteen independent variables were included in the model: tooth type, tooth pathology, dental extraction procedure, operator, primary closure, antibiotic post-extraction, target volume, total dose, timing of extraction post radiotherapy, bony changes in mandible, visibility of upper cortical line of mandibular canal, visibility of lower cortical line of mandibular canal, widening of periodontal ligament space and lamina dura continuity. The analysis with less than 14 variables did not have much effect on the results.

The final model consisted three variables which were the predictors of ORN following a dental extraction (Table 5). The confounding variables from the patients’ factors (Table 1) could not be included into the model to have it adjusted as the total number was different.

**Table 5:** Unadjusted association between dental and radiographic-related factors and ORN

Dental and radiographic factors	OR (95%CI)	*p* value
**Timing of extraction post RT**		
≤ 5 years	1.00	
> 5 years	0.06 (0.01–0.25)	< 0.001
**Extraction procedure**		
Simple extraction	1.00	
Surgical removal	6.50 (1.37–30.91)	0.02
**Mandibular canal UCL visibility**		
Line visible	1.00	
Line not visible	9.47 (1.61–55.88)	0.01

Hosmer–Lemeshow test (X^2^(8) = 5.36, *p* = 0.72); Classification table: % correctly classified = 95.3%, sensitivity = 65.6%, specificity = 98.0%

The odds of developing an ORN from a surgical procedure was 6.50 (CI 1.37–30.91, *p* = 0.02). In addition, dental extraction of more than 5 years after radiotherapy and invisible upper cortical line of the mandibular canal on the DPT had the odds of developing an ORN of 0.06 (CI 0.01–0.25, *p* < 0.001) and 9.47 (CI 1.61–55.88, *p* = 0.01), respectively.

## Discussion

The prevalence of ORN following a dental extraction shown in this study was higher (21.9%) compared to other studies [7, 8, 30, 31]. One of the reasons could be due to the time frame in the definition used in this study which was three months of bony exposure following the dental extraction [2, 24] whereas some studies used six months or one year post dental extraction as the criterion of ORN [32–34]. Delayed wound healing in post-radiation tooth socket is expected [2], however, waiting beyond three months to reassess the bony exposure prior to offering treatment or intervention did not seem to be a good clinical practice [35]. The high prevalence could also be due to the significant portion of the study population had to undergo surgical extraction, which could have contributed to an increased risk of developing ORN. Other possible explanation is because almost half of the patients in this series had undergone concurrent chemoradiotherapy due to the advanced tumour stage. Previously, Reuther et al. [36] found that in their case series the addition of chemotherapy cause an earlier occurrence of ORN.

This study showed that surgical removal of a tooth was six times more likely to develop ORN post extraction, OR = 6.50 (95% CI 1.37–30.91). This could be due to poor cellularity and vascularity in the irradiated periosteum, hence resulted in non-healing wound [37]. Within the marrow, blood vessels of varying sizes including the inferior alveolar artery were obliterated after radiation [38]. Traumatic, surgical procedure or periosteal stripping further decreased bone vitality and increased the risk of ORN [39, 40]. Sometimes, surgery was inevitable due to grossly decayed or impacted teeth. Whenever possible, minimal tissue manipulation is advocated.

The second predictor was related to the period of post radiotherapy. Dental extraction performed more than five years after RT was associated with a reduction in the likelihood of ORN post extraction, OR = 0.06 (95% CI 0.01–0.25) (*p* < 0.001). Different ORN rate was reported at different time interval in the literature. Nabil and Samman reported the incidence rate of 7.5% within first year post RT, 22.6% at the 2–5 years and 17% after 5 years of RT [2]. In a retrospective cohort study of 5783 subjects, the incidence of ORN was shown to increase steadily after the first-year post RT and the peak was at four years post RT [41]. On the other hand, Marx and Johnson observed that the incidence of trauma-induced ORN had bimodal peak [42]. The first peak was in the first three months post RT. The second rise began at about two years with the peak was at about 5 years post RT. In the same study, Marx and Johnson also found that the perfusion of the irradiated tissue and the state of fibrosis became worse over time [42]. It has been suggested that the indicated extractions after RT preferably were to be conducted in 5–6-month post RT before the progressive tissue fibrosis and loss of vascularity set in to reduce the ORN risk [7, 43]. Our study showed a case of ORN that occured following 30 years of RT, and this supported the radiobiological theory. The findings from this and previous studies therefore may conclusively state that the risk of ORN post extraction is present even after many years of RT [24, 41]. Nevertheless, the overall results of our study, showed the contrast. Besides the radiobiological factors, other factors, such as the local factors play a significant role as the ORN risk contributor. Profound state of poor oral hygiene and xerostomia was documented in this study and this could lead to early dental extraction.

Mandibular canal visibility in DPT in relation to ORN changes was explored in this study. In the normal population, it was found that the visibility was the best at the third molar region, followed by the second molar region and lastly, the first molar’s [17–19]. The upper cortical line (UCL) was less visible compared to the lower cortical line (LCL) [15, 16]. Our analysis revealed 42.3% of the UCL and 82.7% of the LCL were visible on the right body region (mental foramen to proximal of angle of mandible) while on the left side, visibility for the UCL and LCL was 50.9% and 90.9% respectively. Its visibility was higher than the results shown by Pria et al. and Naitoh et al. who reported 36.7–39% and 68.4–70.9% visibility of the upper and lower cortical line, respectively [16, 21]. The visibility of mandibular canal in the digital panoramic image was associated with the cancellous bone density in the alveolar region [14, 21]. We postulated that decrease of cancellous bone density or its resorption might cause mandibular canal to be invisible. Visibility of the mandibular canal was shown to be changed by presence or absence of a tooth. Following a tooth loss, the neurovascular bundles that coursed from tooth apices to mandibular canal disappeared and the opening in the superior wall was subsequently calcified, making the superior wall of the mandibular canal becomes more distinct [44].

The majority of extractions involved posterior teeth (63.7%), and this finding was similar with other studies [6, 30, 34]. It was found that upper and lower molar teeth extraction was statistically significant for the occurrence of ORN. There was one lower incisor’s socket which developed ORN (3.1%) while no ORN at all was observed in the upper incisor extractions. Tong et al. reported that extraction of maxillary teeth had the greatest risk of complication among all teeth with 10.5% risk of ORN [34]. Lye et al. showed that from their 155 tooth extractions, all ORN cases involved a maxillary molar (n = 1, 33%) and mandibular molars (n = 2, 67%) [30]. All subjects in the aforementioned studies were NPC patients. Other studies reported much lower ORN risk involving the posterior teeth, range 0–0.6% [6, 8]. In this study, all tumor sites were located at the posterior regions of the oral cavities. For the NPC, the target volume at least included the posterior maxilla. For oral and oropharyngeal tumors, the posterior mandible was invariably included. This could be one of the reasons that majority of extraction sites that developed ORN were the posterior teeth (n = 31, 96.8%) with 19 teeth (86.3%) within the target volume. This study showed that the extraction of the maxillary and mandibular anterior teeth had negligible ORN risk. Posterior teeth should be treated more conservatively and preserved whenever feasible. Prophylaxis should be considered for the extraction of maxillary and mandibular molars besides atraumatic extraction and antibiotic cover. A regular follow-up should be performed until complete healing is observed.

All extraction sites that developed ORN (n = 22) had the total dose of 60 Gy and above. This shows that total dose is an important risk factor for the development of ORN. The risk is minimal if the total dose is below 60 Gy [2]. Thorn et al. found that majority of patients (96.3%) who developed ORN received 60 Gy and above [4]. With the IMRT, if the jaw is not excluded from the planning target volume, the risk was similar, i.e. the part of the jaw that received total dose of more than 60 Gy predisposed a person to higher risk of ORN [40].

The teeth which were within the target volume were significantly associated with ORN post extraction compared to those outside the target volume (23.0% vs. 1.9%). Thorn et al. reported that of the 80 ORN cases, all but one was seen in the target volume [4]. However, there were studies that reported no ORN for teeth in the target volume or outside the target volume [6, 45].

We found that primary closure of the extraction sockets was significantly associated with ORN in the univariate analysis. The aim of primary closure of extraction socket was to prevent food entrapment in the exposed tooth sockets which could increase the risk of infection. However, the tension from manipulating the soft tissue for approximation may damage the small vessels, hence contributing to poor healing. Tight multiple sutures may also strangulate those fragile vessels. We therefore propose that primary closure of sockets should not be mandatory, especially if it required surgical procedure such as alveolar ridge reduction or flap raising. Closure of sockets with sutures was just to reduce defect size and daily socket irrigation should suffice.

Pertaining to the radiographic change, sclerosis was the most commonly found bone change (45.9%) in relation to a mandibular tooth, followed by mixed radiopaque-radiolucent change (11.9%) and bony resorption (5.7%). Bony sclerosis and bone resorption were reported as post-IMRT mandibular changes in a study [10]. Comparison of the prevalence and site of bony changes with other study was not feasible due to different methodology used [10]. Bony sclerosis post radiotherapy had been described as radiation osteitis [46]. It was due to the radiation damage to osteoblasts, followed by secondary resorption of the bone matrix with bone deposition on the unresorbed trabeculae. New bone formation was evident in the cortical and cancellous bone of the irradiated mandible and this was not observed in the non-irradiated animals [38]. This may contribute to the bony sclerosis appearance in the radiograph.

### Limitations

The retrospective design possessed some limitations. Firstly, missing data was apparent especially in four variables, namely, smoking and alcohol intake, xerostomia, tumour staging, type of radiotherapy and concurrent chemoradiotherapy. Future research with prospective study design is suggested to minimize missing data.

Secondly, only assessment of bony changes in relation to the extracted mandibular teeth were included. Radiographic examination for bony changes following extraction of the maxillary teeth were omitted due superimposition of the neighbouring structures. Positioning error especially when the tongue is not placed against the palate causes the formation of soft tissue shadow below the tooth apices [47]. Nasal cartilages, soft palate and dorsum of tongue formed the hard tissue shadows in the maxillary area [48]. These factors obscured accurate assessment of the bony changes in the maxillary tooth sockets.

Thirdly, DPT for normal bone before radiotherapy were not available for majority of patients (93.12%). Post radiotherapy bony changes therefore could not be confirmed as these features might already be present before radiotherapy.

### Future study

For the assessment of the mandibular canals, a cone beam computed tomography (CBCT) would be a better option for the analysis of the upper and lower cortical lines of the mandibular canal [19]. Following that, to validate this finding, a histological study of the mandibular canal wall structure in the irradiated mandible is advocated.

## Conclusion

In conclusion, the prevalence of ORN following a dental extraction was 21.9%. Dental extraction of more than five years after RT, surgical removal procedure and the upper cortical line of mandibular canal being invisible were the predictors of ORN post extraction. The new knowledge produced from this research was the invisibility of the upper cortical line of the mandibular canal could guide specialist and dental practitioner prior to performing post radiotherapy dental extraction.

## Data Availability

The datasets generated and/or analyzed during the current study are not publicly available because they contain personal information but are available from the corresponding author on reasonable request.

## Abbreviations

ORN: Osteoradionecrosis
DM: Diabetes mellitus
Thyr: Thyroid related disease (hypo/hyperthyroidism)
DPT: Dental panoramic tomogram
RT: Radiotherapy
Gy: Gray
MC: Mandibular canal
UMMC: University Malaya Medical Centre
UKMMC: Universiti Kebangsaan Malaysia Medical Centre
FDUM: Faculty of Dentistry, University of Malaya
PACS: Picture archiving and communication system
kVp: Kilovolt peak
mA: Milliampere
WPLS: Widening of the periodontal ligament space
SD: Standard deviation
NPC: Nasopharyngeal carcinoma
SCC: Squamous cell carcinoma
AJCC: American joint committee on cancer
UCL: Upper cortical line
LCL: Lower cortical line
OR: Odds ratio
CBCT: Cone beam computed tomography
IMRT: Intensity-modulated radiation therapy
